# Music-based interventions in the feeding environment on the gut microbiota of mice

**DOI:** 10.1038/s41598-023-33522-3

**Published:** 2023-04-18

**Authors:** Junyi Niu, Hongli Xu, Guosheng Zeng, Pengpeng Wang, Bakint Raciheon, Shah Nawaz, Zhibo Zeng, Jiewei Zhao

**Affiliations:** 1 College of Music and Dance, South-Central Minzu University, Wuhan, 430074 People’s Republic of China; 2 College of Veterinary Medicine, Huazhong Agricultural University, Wuhan, 430070 People’s Republic of China; 3 Institute of Agricultural Sciences, ETH Zurich, Universitaetstrasse 2, 8092 Zurich, Switzerland; 4 People’s Government of Shian Town, Nanyang City, 473540 Henan Province People’s Republic of China

**Keywords:** Microbiology, Health care, Astronomy and planetary science

## Abstract

Gut microbiota is established to be associated with the diversity of gastrointestinal conditions, but information on the variation associated with music and gut microbes is limited. Current study revealed the impacts of music intervention during feeding on the growth performance and gut microbes of mice by using clinical symptoms and 16S rRNA sequencing techniques. The results showed that feeding mice with music had a significant increase in body weight after the 25th day. The *Firmicutes* and *Proteobacteria* were the most dominant phylum in the gut microbiota. Also, the relative abundance of the dominant bacteria was variable after musical intervention. In contrast to the control group, a significant decrease in alpha diversity analysis of gut bacterial microorganisms and Metastats analysis showed a significant increase in the relative abundance of 5 genera and one phylum after the music intervention. Moreover, the musical intervention during feeding caused modifications in the gut microbial composition of mice, as evidenced by an increase in the level of *Firmicutes* and *Lactobacillus*, while decreases the richness of pathogenic bacteria, e.g. *Proteobacteria*, *Cyanobacteria* and *Muribaculaceae*, etc. In summary, music intervention increased body weight and enhanced the abundance of beneficial bacteria by reducing the prevalence of pathogenic bacteria in gut microbiota of mice.

## Introduction

Gut microbiota (GM) is a diverse population of microbes that inhabitant in the host digestive system^1^. Animal gut microbiota form an ecosystem, and the healthy animals GM prevails in a dynamic balance^2^. Abundant, active, and stable GM promoted human health, and new evidence revealed its an indispensable role in the human body^3^. The number of microorganisms in the gut is enormous, up to one hundred trillion^4^. The type, distribution, function and characteristics of the GM vary in different animals and even in different ages of the same individual^5^. An inevitablelinkage was revealed between centeral nervous system (CNS) and gut microbiota^6^. The theory of the gut-brain axis, suggests that the CNS-regulated gut microbiota to influences brain activity^7^. A healthy GM can positively regulate the neuroimmune response to the CNS; conversely, the dysregulation of GM increased the risk of neurodegenerative diseases and exacerbated the disease response to neurological disorders, which in most cases were accompanied by psychiatric, psychological abnormalities and disorders of the GM^8–11^.

Music, the sound wave stimulus, affected the physiology and psychology of animals. Previous studies have demonstrated that feeding animals with music can affect their growth performance and animal production^12^. The rhythm of music encouraged muscle activity and induced vitality in the organism. When the rhythm of music is close to the heart rhythm of the organism, it stimulates the organism to excrete regulating hormones^13^. A study with music therapy was developed in the United States of America, revealed that it could be applied to unhealthy and injured people to relieve emotions, improve digestion, balance the mind stats, and promotes recovery from disease^14^. Since China introduced the five-elements music therapy (FEMT) into the medical field in the Pre-Qin Dynasty, the Chinese FEMT had a long history and a complete system^15^. Scientists observed that FEMT could relieve anxiety symptoms, improve spatial cognition, regulate intestinal microbiota, and assist in drug therapy^16^. It was hypothesized that the interaction of the bidirectional pathways between the CNS and the gastrointestinal system influenced primarily by the GM, indicating the importance of GM in the treatment of neurological diseases^17^. Although there are limitations in the mechanisms of music therapy regarding the treatment of disease at present, music therapy was used as a common assistant clinical treatment^18^. However, there are few reports on the effects of listening music during the feeding process on GM. Therefore, the primary objective of this study was to evaluate the effect of feeding with music on GM and growth performance in mice to provide theoretical support for music therapy.

## Results

### Clinical symptoms of animal experiments

**Figure 1:**
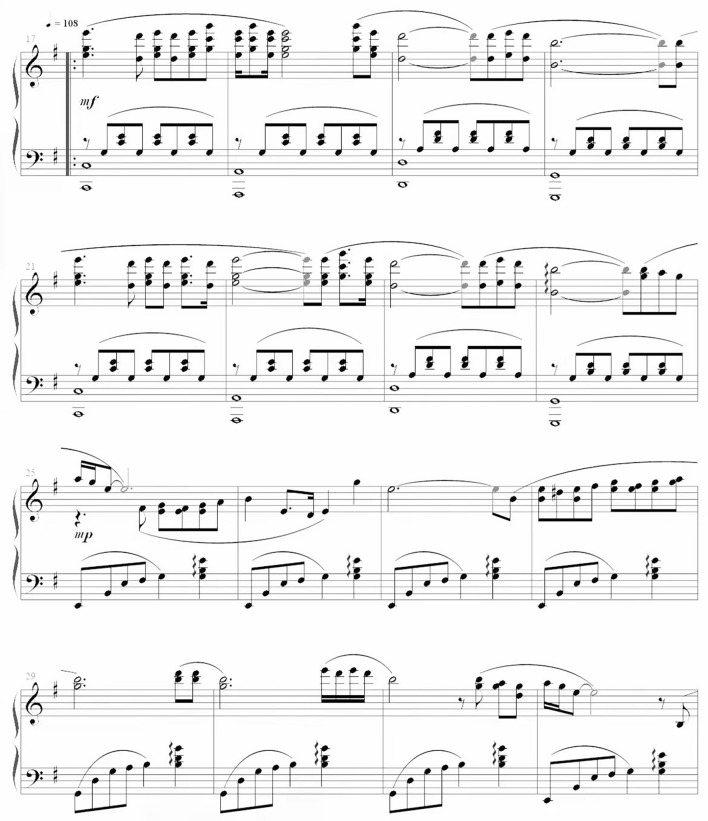
Music played was a compilation of New Age songs. As the figure is music selection of the El Condor Pasa, *DanieL alomia robles*as. The Jm groups were kept environment as above music for 6 h at 10:00 am every day throughout 30 days.

No animal suffered from depression, illness, or passed away during the entire study. Neither necropsy nor electron microscopic imaging revealed macroscopic and microscopic pathologies. The mice weight addition is shown in Table 1. The weight of the CG group was higher than the MG group (music intervention during feeding, Fig. 1) during the initial 16 days out of the 30-days feeding period, with a significant difference between the two groups on the 4th day (*P* < 0.05). However, the mice weight between the two groups was overturned after the 19th day, the weight of MG group was higher than that of the CG group between days 19–30 days. Among them, on 25th (38.444 versus 39.600), 28th (39.120 versus 40.238) and 30th (40.120 versus 40.838) day, there was a statistical difference between the groups (*P* < 0.01; *P* < 0.05; *P* < 0.05). Interestingly, during the feeding period we found that the mice following the music intervention were more active than the control mice regarding their emotional condition and activity status.

**Table 1:** The mice weight throughout the experiment period.

Day (d)	Weight (g)		SEM	*P*-value
	CG	MG		
1, d	27.154	26.706	0.497	0.6786
4, d	31.060	29.262	0.462	0.0424*
7, d	26.500	26.456	0.306	0.9476
10, d	32.902	32.576	0.368	0.6836
13, d	28.626	28.256	0.345	0.6216
16, d	29.896	29.458	0.417	0.6285
19, d	31.656	31.966	0.404	0.4260
22, d	34.146	35.638	0.516	0.1587
25, d	38.444	39.600	0.148	0.0042**
28, d	39.120	40.238	0.269	0.0318*
30, d	40.120	40.838	0.187	0.0464*

The data were expressed as the mean, SEM (standard error of mean) and *P* value (*P < 0.05; **P < 0.01).

*CG* control group, *MG* musical intervention group.

### DNA sequence analyses

**Figure 2:**
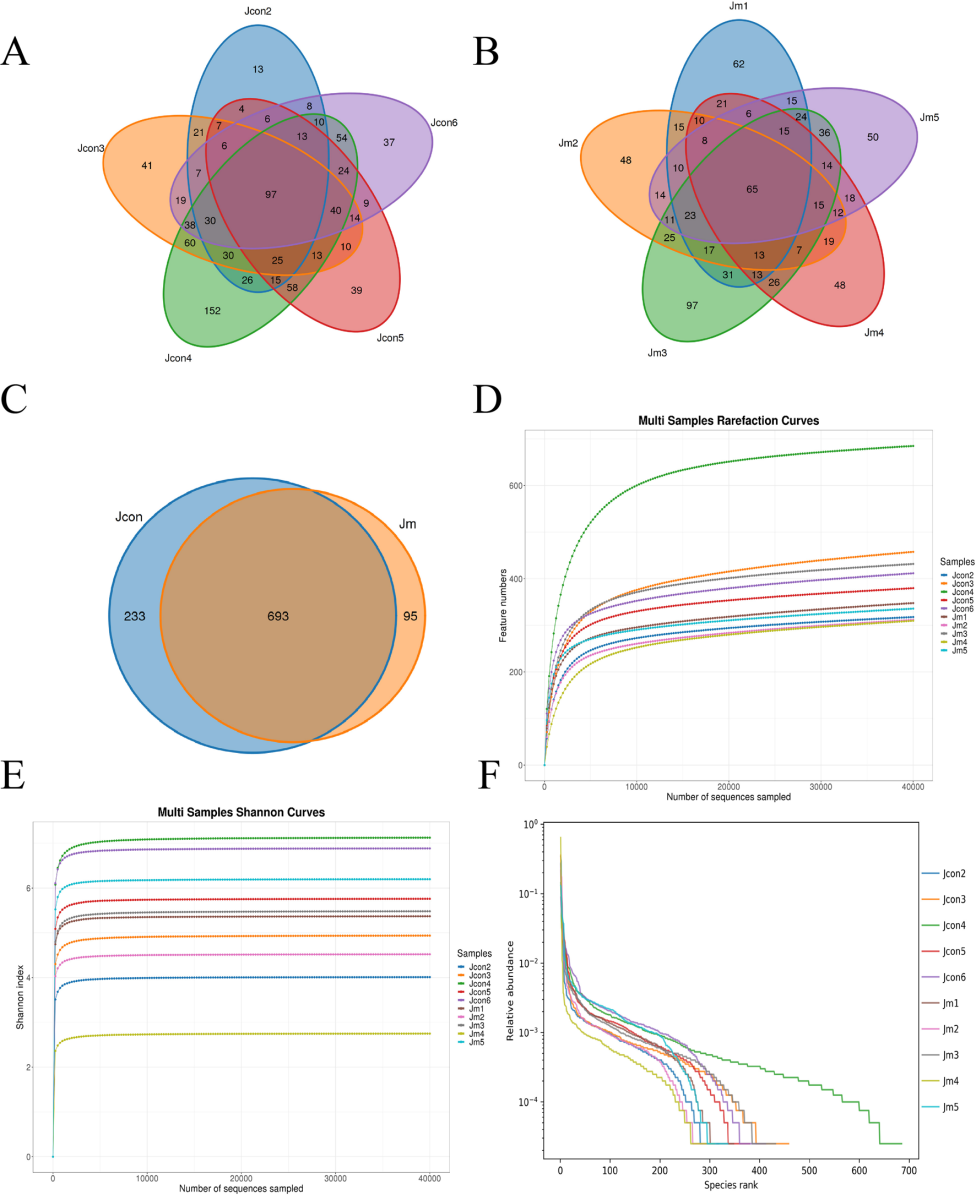
Operational taxonomic units (OTUs) and sample feasibility analysis. (**A**) The total number of OTUs in Jcon groups (Jcon2, Jcon3, Jcon4, Jcon5 and Jcon6); (**B**) the total number of OTUs in Jm groups (Jm1, Jm2, Jm3, Jm4 and Jm5); (**C**) venn diagrams show specific and common bacteria OTUs in Jcon group and Jm group; (**D**) rarefaction curves; (**E**) rank abundance curves; (**F**) species accumulation curve.

Following microbial composition assignment, a total of 400,333 and 399,471 primitive sequences were found from Jcon and Jm groups that based on 97% nucleotide sequence similarity (Table 2). After removing the unqualified reads, a total of 797,069 reads were available in this study, including Jcon 398,941 and Jm 398,128 reads. The total effective reads used for resulting analysis were 776,089, with an average of 77,609 per sample, ranging from 75,336 to 78,401 reads of all samples. There were 1021 OTUs detected in this test (Fig. 2C), among which Jcon (Fig. 2A) had 926 OTUs (Jcon2 has 318 OTUs; Jcon3 had 458 OTUs; Jcon4 had 685 OTUs; Jcon5 had 380 OTUs and Jcon6 had 412 OTUs) and Jm (Fig. 2B) had 788 OTUs, among them Jm1 had 348 OTUs, Jm2 had 458 OTUs, Jm3 had 432 OTUs, Jm4 had 310 OTUs and Jm5 had 336 OTUs, among which the two groups commonly have 693 OTUs. The Feature curve (Fig. 2D) and Shannon (Fig. 2E) curve tended to be horizontal and stable characteristics, indicating that the sequencing volume and depth justify the analysis requirements. Furthermore, the relative abundance curve was wide and decreased gently, showing significant uniformity and abundance (Fig. 2F).

**Table 2:** Sample sequencing data results statistics.

Sample	Raw reads	Clean reads	Effective reads	Average length (bp)	Effective (%)
Jcon2	79,824	79,533	78,372	418	98.18
Jcon3	79,882	79,597	77,450	422	96.96
Jcon4	79,934	79,654	75,336	419	94.25
Jcon5	80,379	80,116	78,401	418	97.54
Jcon6	80,314	80,041	78,373	418	97.68
Jm1	79,865	79,592	78,296	420	98.04
Jm2	79,937	79,644	78,033	421	97.62
Jm3	79,767	79,523	75,783	418	95.01
Jm4	79,869	79,609	77,981	427	97.64
Jm5	80,033	79,760	78,064	421	97.54

### Alteration of gut microbial diversity

**Figure 3:**
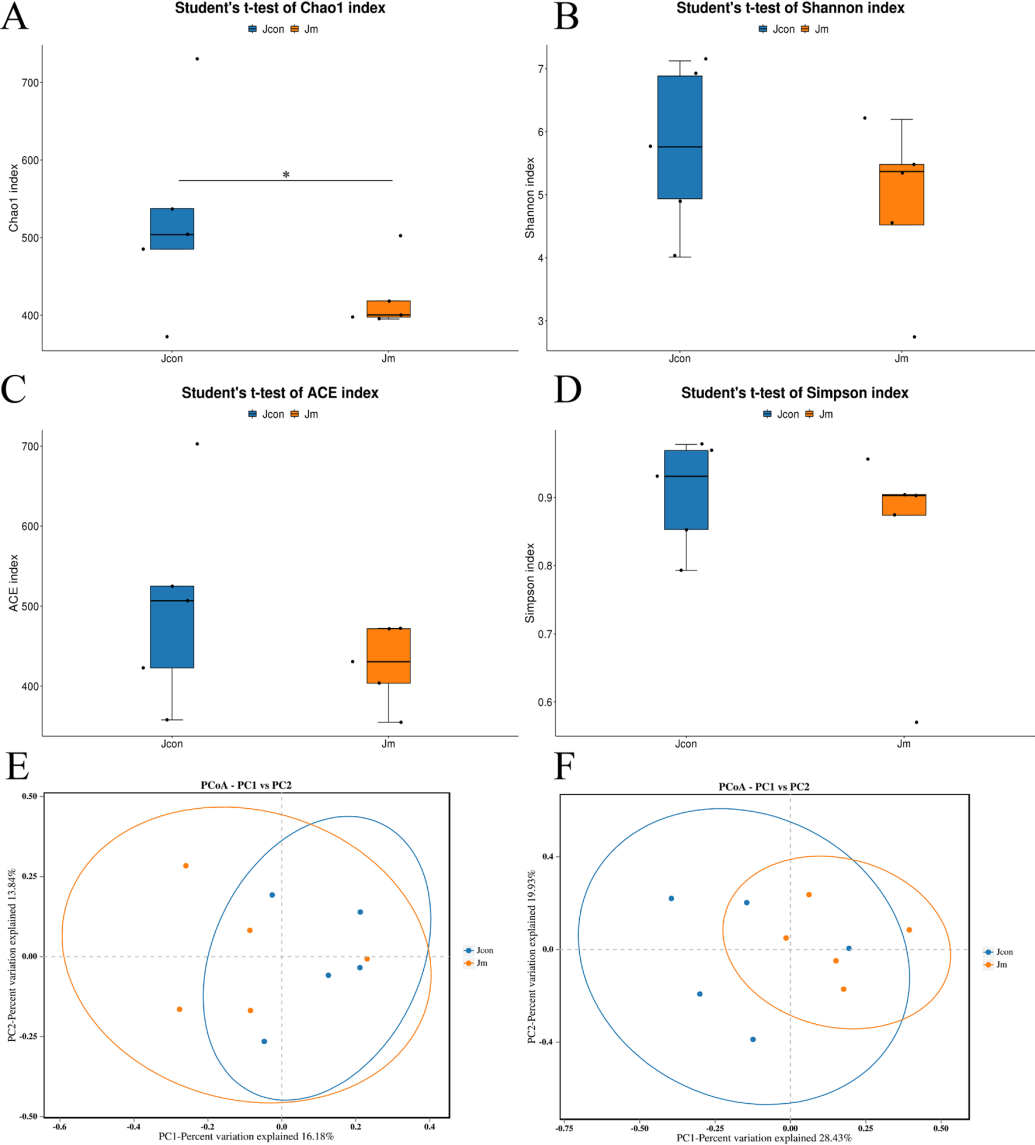
Alpha (α) and Beta (β) diversity analysis. (**A**) Chao1 index; (**B**) Shannon index; (**C**) ACE index; (**D**) Simpson index. The PCoA analysis showed different of Jcon and Jm group. (**E**) Unweighted—binary jaccard analysis; (**F**) weighted—bray curtis analysis.

We matched the qualified sequences gained by seizure and performed α-diversity analysis to assess the differences in the diversity and abundance of intestinal communities between control and music intervened groups. The results showed that the microbial diversity and abundance were higher in the Jcon group than that of Jm, with a significant chao1 (Fig. 3A) index difference among both groups (*P* < 0.05). The other three indices (Shannon index, ACE index and Simpson index), didn’t show statistical differences (Fig. 3B–D). Taken together, it could be seen that the feeding process with music decreases the abundance of intestinal microflora. The β diversity analysis was calculated using QIIME software to compare the similarity in the variety of species diversity between samples. The PCoA analysis was based on the unweighted—binary jaccard (Fig. 3E, *P* > 0.05) and weighted—bray curtis (Fig. 3F, *P* > 0.05) showed no statistical difference between the two groups. The results showed no difference, indicating a high similarity and low species diversity among both groups.

### Microbial composition of the mouse intestines

**Figure 4:**
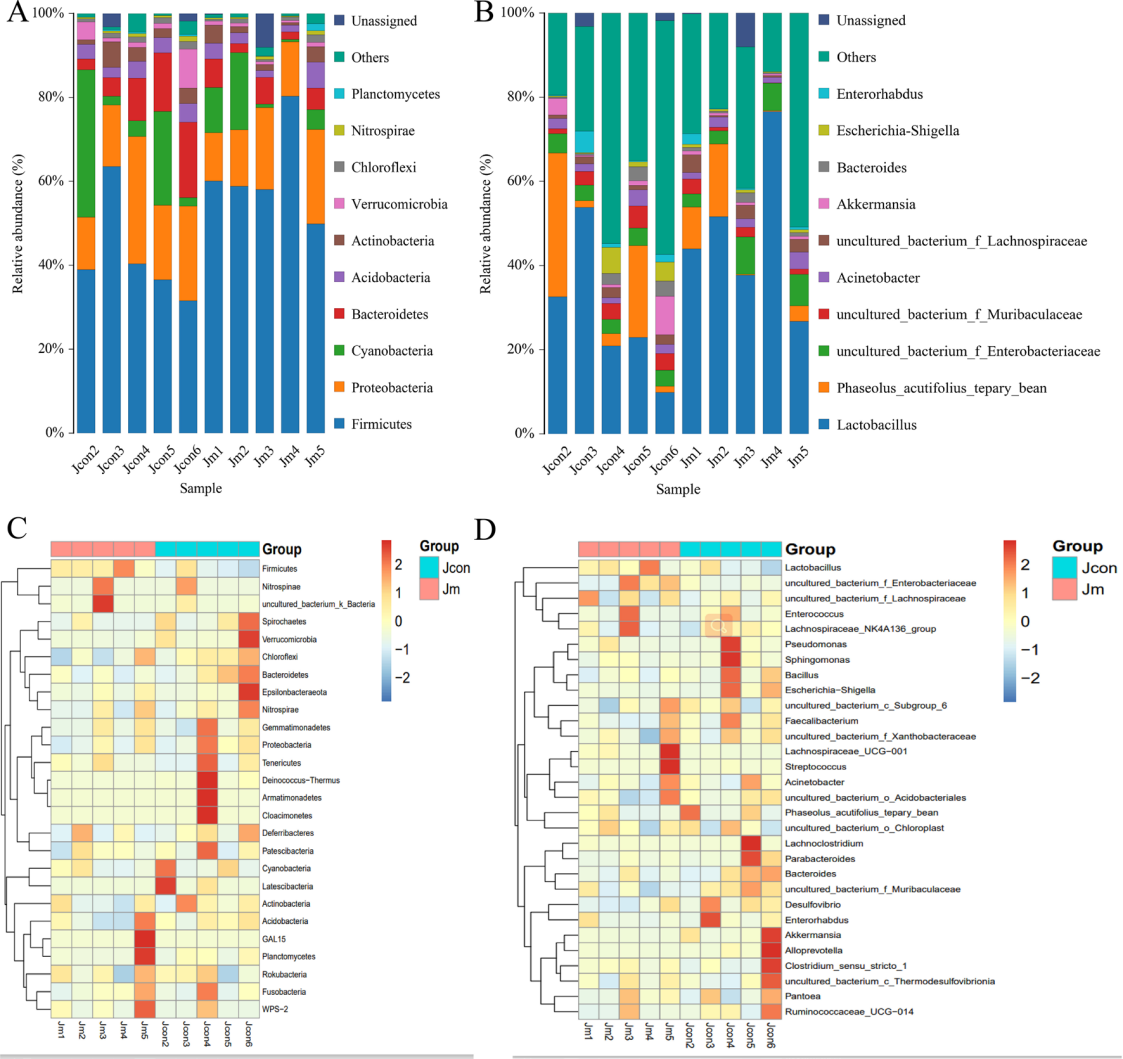
Mice gut microbial composition distribution and heat map. (**A**) The dominant bacterial species and abundance at the phylum level; (**B**) genus level. (**C**) Heat map of microbial similarity between samples at the phylum level; (**D**) genus level.

The relative proportions of dominant samples at the phylum and genus levels were assessed by classifying microbial taxa from different species using QIIME2 software. The results showed that the dominant bacteria at the phylum level in the Jcon were *Firmicutes* (42.19%), *Proteobacteria* (19.57%), *Cyanobacteria* (13.05%) and *Bacteroidetes* (9.85%), which accounted for 84.66% of the gut microbiota; in Jm, the dominant bacteria at the phylum level were *Firmicutes* (61.42%), *Proteobacteria* (16.00%), *Cyanobacteria* (7.07%) and *Bacteroidetes* (4.46%), which accounted for 88.94% of the microorganisms (Fig. 4A). The microbial composition (genus level) of both groups showed *Lactobacillus* as the dominant community. Besides, *uncultured_bacterium_f_Enterobacteriaceae* (3.97% versus 5.83%) and *uncultured_bacterium_f_Muribaculaceae* (3.47% versus 1.62%) were further dominant strains in both groups (Fig. 4B). From the above results, it could be concluded that musical intervention during the feeding of mice could alternate the composition of intestinal microorganisms. The heat map indicates the similarities and differences in the gut microbial papulation of multiple samples through the color ramp and degree of closeness (Fig. 4C,D). The results showed that low similarity in microbial abundance between samples of the two groups both at the bacterial phylum level (Fig. 4C) and at genus level (Fig. 4D), but less variation between samples within the groups and high similarity in microbial abundance.

**Figure 5:**
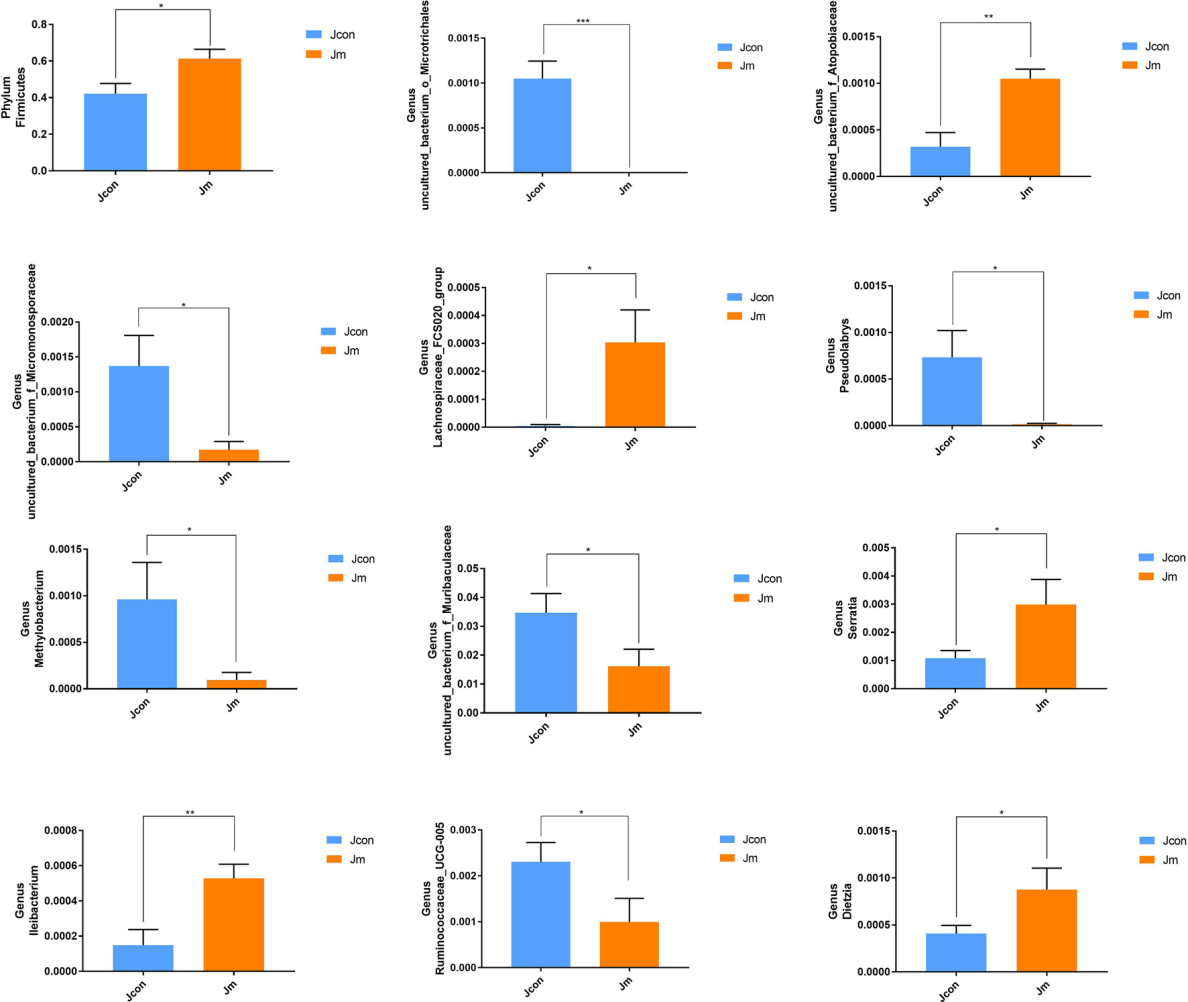
Metastats analysis. The statistical differences in the gut bacterial abundance at the level of phylum and genus. All of the data represent means ± SD (*P < 0.05; **P < 0.01; ***P < 0.001).

**Figure 6:**
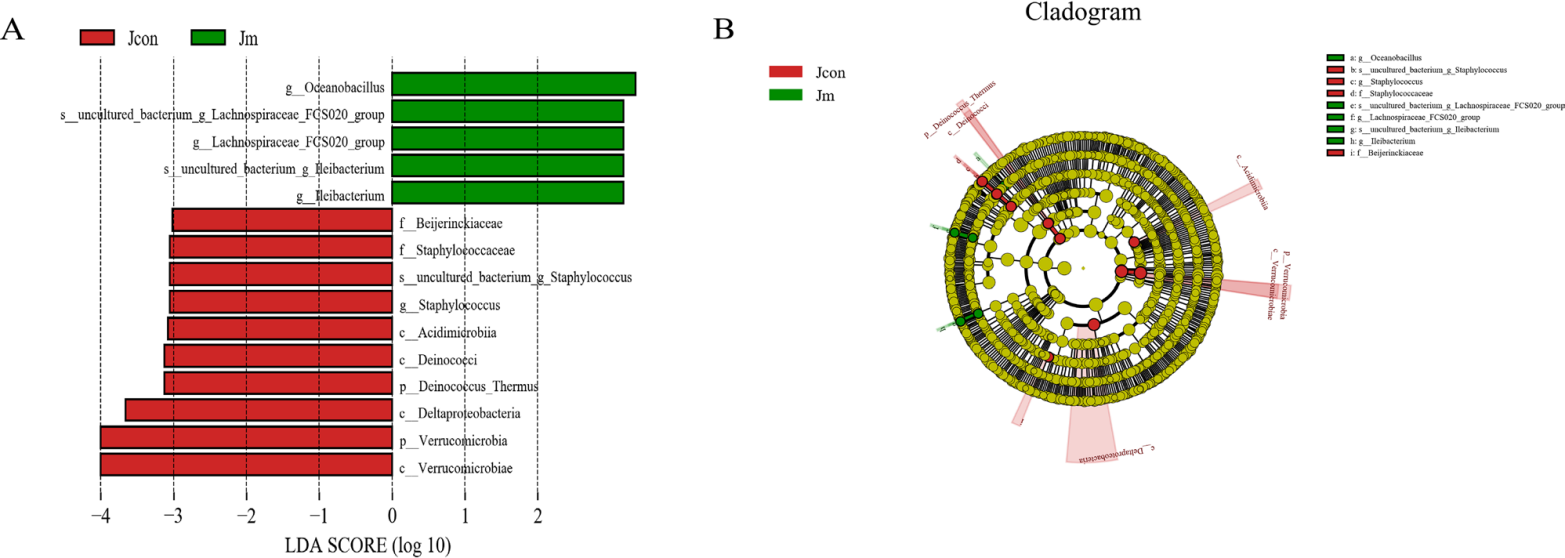
The combined LEfSe analysis and LDA score revealed statistically significant differences in bacterial abundance. (**A**) LDA scores > 3.0 were considered statistically significant. (**B**) Cladogram depicted the phylogenetic distribution of gut microbiota associated with the Jcon and the Jm group.

Using Metastats analysis, statistically different classifications of phylum and genus level composition of the two groups of microorganisms were investigated (Fig. 5). The results showed significant differences in relative abundance of *Firmicutes* (Jcon 0.422 ± 0.056 versus Jm 0.614 ± 0.051, *P* < 0.05) at the phylum level. Moreover, eleven statistically significant taxa at genus level were found, where Jcon was higher in *uncultured_bacterium_o_Microtrichales* (*P* < 0.0001), *uncultured_bacterium_f_Micromonosporaceae* (*P* < 0.05), *Pseudolabrys* (*P* < 0.05), *Methylobacterium* (*P* < 0.05), *uncultured_bacterium_f_Muribaculaceae* (*P* < 0.05) and *Ruminococcaceae_UCG-005* (*P* < 0.05) than in the Jm group. Contrary to this, the Jm was higher in *uncultured_bacterium_f_Atopobiaceae* (*P* < 0.01), *Ileibacterium* (*P* < 0.01), *Lachnospiraceae_FCS020_group* (*P* < 0.05), *Serratia* (*P* < 0.05) and *Dietzia* (*P* < 0.05) than the Jcon group. Considering the limitations of the Metastats analysis for difference in bacterial relative abundance between two groups, the LEfSe analysis (Fig. 6) was performed to search for statistically different biomarkers between Jcon and Jm. The results showed that fifteen statistically different biomarkers were recovered, in addition to the above mentioned significantly different bacteria, that the most dominant groups in Jcon were *Verrucomicrobia*, *Deltaproteobacteria* and *Acidimicrobiia*, while *Lleibacterium* and *Oceanobacillus* were significantly expressed in Jm group.

### Web-based correlation analysis

**Figure 7:**
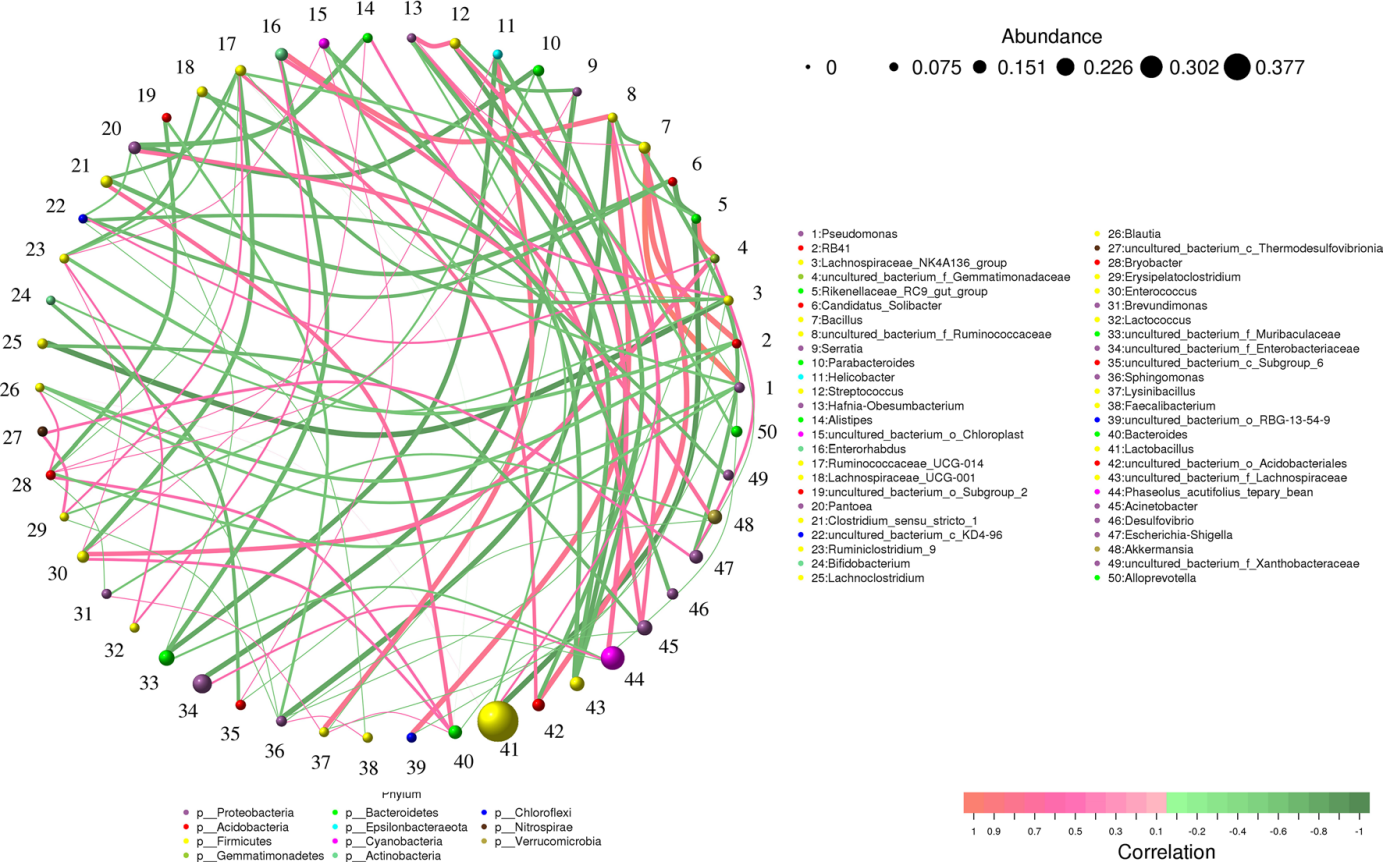
Analysis of correlations between species. The lines depicted the correlation between two species, and the line thickness depicted the strength of the correlation. According to the line's color, orange denotes a positive correlation, and green, a negative correlation.

In the field of ecology, correlations are often used to construct network models that could be implemented to analyze community data of species (co-occurrence pattern) or to combine multiple data sets for analysis. The taxonomic analysis of all species in this study was shown in supplementary information (Table S1). Top 50 genera were used as base on python to create a web-based correlation, which indicated 78 nodes, 237 edges and 6 communities (Fig. 7). We found that *Lactobacillus* was the most abundant bacteria among the two groups regarding web-based correlation analysis. Besides, abundance of *Lactobacillus* in mouse after music intervention (Jm group) was higher than that of control group (47.34% versus 28.03%), while the *Phaseolus_acutifolius_tepary_bean* richness was lower than control group (6.24% versus 12.36%). The *Lactobacillus* showed negative correlation with *Parabacteroides* (0.6364), *Faecalibacterium* (0.7455), *Helicobacter* (0.7720), *RB41* (0.6485), *Blauti* (0.8182) and *Staphylococcus* (0.7538). The *Phaseolus_acutifolius_tepary_bean* showed positive correlation with *Candidatus_Koribacter* (0.7173) and *Breznakia* (0.7195), while it showed negative correlation with *Pantoea* (0.66060), *Enterococcus* (0.6809) and *Ruminococcaceae_UCG-014* (0.6606).

## Discussion

In recent years, increasing attention has been absorbed on environment and animal welfare-associated research^19^. The enrichment and diversity of environmental factors was an important research parameter to improve animal welfare^20^. The environmental factors referred to a normal environment in which animals have been provided with the environmental incentives to make beneficial enhancements that allow them to express their behavior and mental activities normally, improving health status and growth performance^21,22^. Music, as an emotion-reflecting art, could play a positive role in reducing stress in animals^23^. The hypothalamic–pituitary–adrenal (HPA) axis is an important part of the neuroendocrine system that plays a vital role in regulating the stimulation response of the animals, and its activity could reflect the stress intensity of the animals, in addition, corticosterone (Cort) is a marker of the excitability of the HPA axis^24^. Previous studies have shown that a mixture of music and inspected sounds significantly reduces the feed-to-meat ratio of piglets and improves their growth performance. However, there are some music (e.g. heavy metal, frequency < 20 Hz, 95–105 dB) that have the opposite effect^25,26^. This study was performed on El Condor Pasa music, which was natural in style and not overly layered^27^. In the 30-days feeding experiment (with music intervention), it was found that the body weight of the mice was significantly higher than control group at day 25. Moreover, Gao et al.^28^ also revealed that the animals consumed more diet and gained significantly more weight after the musical intervention. Some research has found that music can promote immune systems and gut microbial nutrition absorption while also mitigating the negative impacts of noise^29^. Overall, this study indicated that the music could improve body weight and promote the growth and physical development of mice.

Jejunum is the primary digestive and absorption site of animals, it is essential for animal growth and development^30^. The nutrients and water in the jejunum pass through the intestinal mucosal epithelium, eventually enter the blood and lymph^31^. Among them, the intestinal mucosal epithelium is composed of simple columnar epithelial cells, goblet cells and a few endocrine cells^32^. However, research shows that the absorption of these substances could not be achieved with jejunal mucosal epithelium but the jejunal microbiota^33^. Music have been found to have a positive effect on the body, inducing a coordinated resonance and promoting the harmony of organ rhythms, along with a series of endocrine transformations^34^. Furthermore, it influence the coordinated resonance of the digestive system, the secretion of growth hormones, and the growth of beneficial bacteria^35^. The analysis of DNA sequences of GM showed that the number of OTUs and Reads of music group were lower than those of the control group, indicating that the musical intervention at feeding environment reduced the abundance of microorganisms in the intestinal habitat. Moreover, the mice GM α-diversity and PCoA analyses showed that the music group was modified in comparison to the control group. Interestingly, the whole diversity analysis revealed a high aggregation of intra-group samples in music group and a significant enhancement of intra-group similarity. Sangkyu et al. suggested that a decrease in gut microbial diversity was associated with a decrease in specific bacteria or overgrowth of individual strains^36^. This showed that musical intervention during feeding can reduce discrete organism microbiota and contribute to the relative stability.

Gut microbiota, the microbial community that resides in the animal intestine, an increasing research trend have been observed in gut microbiota associated fields e.g. microbiology, medicine and genetics, etc^37^. Analysis of the gut microbiota in the Jcon and Jm groups revealed that *Firmicutes*, *Proteobacteri*, *Cyanobacteria*, *Bacteroidetes* and *Lactobacillus* were the most dominant bacteria at the phylum and genus level. Homeostasis of the gut microbiota found to be an essential barrier for resident invasion and colonization by external disease agents, and alterations to the gut microbiota may be linked to a variety of diseases^38^. Behera et al. indicated that the occurrence of the disease was inextricably linked to the GM^39^. Apart from gastrointestinal diseases and metabolic diseases, the intestinal microbiota was also associated with a variety of systemic diseases, such as neurological, respiratory, cardiovascular, and oncological diseases^40^. Moreover, previous studies have shown that FEMT reduced depression and enhance the activity and diversity of the intestinal flora^41^. We found that musical interventions during feeding, statistically increased the richness of *Firmicutes* and *Lactobacillus*, while exponentially reduced the production of *Cyanobacteria*, *Bacteroidetes*, *Phaseolus_acutifolius_tepary_bean* and *uncultured_bacterium_f_Muribaculaceae* in intestinal microorganisms. Talib et al. revealed that *Firmicutes* and *Lactobacillus* were predominant bacteria, indicating a good intestinal health condition^42^. Furthermore, the Metastats analysis (*P* < 0.05) with two groups showed significant differences at the genus level with an extra 11 bacterial species, namely: *uncultured_bacterium_o_Microtrichales*, *uncultured_bacterium_f_Micromonosporaceae*, *Pseudolabrys*, *Methylobacterium*, *uncultured_bacterium_f_Muribaculaceae*, *Ruminococcaceae_UCG-005*, *uncultured_bacterium_f_Atopobiaceae*, *Ileibacterium*, *Lachnospiraceae_FCS020_group*, *Serratia* and *Dietzia.*

*Firmicutes* were the most numerous bacterium, most of which are Gram-positive and appear as spherical or rod-shaped, and many members of *Firmicutes* are beneficial bacteria such as *Lactobacillus*, *Bacillus*, *Bifidobacterium*, *Clostridium butyricum*, etc^43^. It has been shown that microorganisms such as *Lactobacillus* could produce acetate (a short-chain fatty acid), lactate and antibacterial substances that can prevent pathogens from interfering with health^44^. Moreover, they can help in maintaining the micro-ecological balance of the intestines, prevent and inhibit the occurrence of tumors, enhance animal immunity, promote digestion, synthesize amino acids and vitamins^45^. Interestingly, it was found that feeding with music could increase the papulation of *Lactobacillus*. Additionally, it was also found that feeding with in the presence of music caused significant differences in some bacteria among the intestinal microbiota of mice, indicating that such variations may play an important role in the intestinal ecosystem and function. Studies showed that increased gene levels of *Lactobacillus* in the intestinal microbiota promote growth in mice, trigger the production of interferon and enhance the resistance to animal disease^46^. Moreover, LEfSe analysis revealed the recovery of 15 statistically different biomarkers among two groups. Specifically, the relative abundance of 5 bacteria significantly increased in the music group, and the relative abundance of 10 bacteria (*Beijerinckiaceae*, *uncultured_bacterium_g_Staphylococcus*, *Staphylococcaceae*, *Staphylococcus*, *Acidimicrobiia*, *Deinococci*, *Deinococcus_Thermus*, *Deltaproteobacteria*, *Verrucomicrobia*, *Verrucomicrobiae*) significantly increased in the Jcon group.

## Materials and methods

### Ethical certification

We followed the guidelines of ARRIVE^47^. The animal experiments were performed in conformity with the Committee on Animal Ethics Code of Operations, Huazhong Agricultural University (license number HZAUMO-2022-0011). All methods were conducted in accordance with the relevant guidelines and regulations.

### Animals and sample collection

A total of 20 male SPF KM (Kunming) mice were purchased from the Experimental Animal Center of Huazhong Agricultural University (Permits No: SYXK 2020-0084), and all mice weighed 18.0 ± 2.0 g (4 weeks old) and were raised in the Experimental Animal Center (Wuhan, China). During the entire feeding period, sufficient diet (LAD3001G, TROPHIC Animal Feed High-Tech Co. Ltd, China) and water (distilled water) were guaranteed ad libitum for all mice. Moreover, the 12 h of normal light, room temperature of 24.0 ± 2.0 °C, and suitable humidity in the feeding environment was provided. Acclimatization feeding for 2 days in the above-mentioned feeding environment. Subsequently, mice (30 days old) were randomly divided into 4 cages with 5 mice per cage marked as Jcon, Jcont, Jm and Jmt groups. The mice in the Jcon and Jcont groups (CG group) were reared in a normal environment; the Jm and Jmt groups (MG group) were fed in the same environment except for 6 h of music (El Condor Pasa, *DanieL alomia robles*as shown in Fig. 1)^48^ at 10:00 am every day throughout 30 days. Day 1 after the acclimation feeding completed had seen the start musical intervention. Throughout the feeding period, we employ the clinical symptoms to detect the physical signs and survival rate, the mental status was tested with open field test (OFT) method and daily body weights were recorded. After 30 days of feeding, mice were processed with the cervical dislocation method, and the mice were necropsied with reference to the *Lorna*’s method^49^. The jejunum and jejunal contents of the mice in the Jcon and Jm groups were taken immediately into sterile 1.0 mL lyophilized tubes and stored at − 80 °C for microbiological studies. To investigate the effect of musical intervention in the mice growth environment on the CNS, the hippocampus and jejunum of the Jcont and Jmt groups were cleaned with sterile saline and then stored frozen at − 80 °C. Fresh tissue samples from both groups (Jcont and Jmt groups), including the hippocampus and jejunum, were immediately fixed in 4% paraformaldehyde at ambient temperature. The fixed tissues were conducted with the assistance of the company (Pinuofei Biological Technology Co, Wuhan). Tissue samples were evaluated under an electron microscope to assess histological changes.

### DNA extraction and sequencing

Extraction of microbial genomic DNA from jejunal contents samples were used in the FOREGENE DNA Mini Kit (Chengdu, China) according to the recommended guidelines. The recovered DNA was electrophoresed on a 0.8% agarose gel to confirm completeness and magnitude, and DNA concentration was calculated using a UV–visible spectrophotometer (Alpha-1506, China). Amplification of 16 s rRNA V3/V4 to the conserved region by polymerase chain reaction (PCR) using bacterial general primers 338F (50-ACTCCTACGGGAGGCAGAG-30) and 806R (50-GGACTA CHVGGGTWTCTAAT-30). Amplified PCR products were extracted with QIAquick Gel Extraction Kit (Qiagen, USA) for recovery of target sequences. Based on the preliminary ionization results of electrophoresis, the purified PCR products were fluorescently determined on a Microplate reader (PHERAstar FSX, Germany). Afterward, following the fluorescent quantification and sequencing analysis quality measurements, each sample was blended in the appropriate ratio. Purified PCR was used to produce sequencing libraries using Illumina TruSeq (Illumina, United States) according to the manufacturer's statement. Spliced and screened only single peaks and raw data at concentrations ≥ 2 nM, filtering out polluted data such as chimeric sequences, nucleotide mismatches, and indistinct reads to get exact and reliable sufficient data.

### Bioinformatics and data analysis

The Trimmomatic (0.33, https://github.com/usadellab/Trimmomatic) was utilized to filter raw data based on the quality of single nucleotides. Cutadapt (1.9.1, http://cutadapt.readthedocs.org/) was used to identify and remove primer sequences. The USEARCH (10.0.240, http://drive5.com/usearch) was used to construct the PE readings collected in earlier phases, which were then chimera removed using UCHIME (4.1, http://drive5.com/uchime/uchime_download.html). Classify-consensus-blast in QIIME2 (2020.6.0, https://qiime2.org) was a blast-based annotation technique, as the name suggests. It found the annotation with the greatest consensus among N outstanding hits. USEARCH (10.0.240, http://drive5.com/usearch) was used for four main parts that were 97% similar and the conservative OTU filtration cutoff is 0.005%. All sequences were divided into OTUs for various levels of similarity among samples, and each OTU corresponds to one representative sequence. Alpha diversity (Chao1, Ace, Shannon and Simpson) reflected the abundance and diversity among species, and its analysis was performed on QIIME2 (2020.6.0, https://qiime2.org) software. The PCoA analysis was based on the unweighted—binary jaccard and weighted—bray curtis to assume that there exist data that could measure the difference or distance between samples, the approach generated a rectangular coordinate system. Moreover, the T-tests on microbial diversity statistics comparing two groups were performed using Metastats (http://metastats.cbcb.umd.edu/), as the species richness is a continuous variable, meaning it is measured on an interval or ratio scale. The non-parametric factorial Kruskal–Wallis (KW) sum-rank test available for LefSe analysis (Lefse 1.1.1 software, https://github.com/SegataLab/lefse/tree/master/lefse). We used plain package in R language (v3.0.3, https://cran-archive.r-project.org/bin/windows/base/old/3.0.3/), Python (3.8.1, https://www.python.org/downloads/release/python-381/) and GraphPad (6.0, https://www.graphpad.com/dl/96314/10B92408/) for network-wide correlation and statistical analysis. The heat map was produced using the pheatmap (1.0.12) software, of which the link was https://cran.r-project.org/web/packages/. The Spearman (default method) rank correlation analysis was performed based on the quantity of each sample bacterium. The correlation network was built using correlations more than 0.1 and p-values less than 0.05. For statistical analysis SPSS 7.0 software (https://en.freedownloadmanager.org/users-choice/Free_Download_Spss_7.0.html) was used for calculations and P-values were labeled.

## Conclusion

Overall, present study described variation of the gut microbiota in mice listening music during feeding. The results showed significant alterations in the gut microbiota after musical intervention, characterized by a decrease in intestinal bacteria diversity and alteration in mice gut microbiota composition. In addition, the papulation of beneficial bacteria increased, while the pathogenic or conditionally pathogenic bacteria were decreased during feeding mice with music. These results contribute in understanding of the relationship between music and the gut microbiota, as well as the essential information that gut microbiota could alter with accordance to different feeding environment. This study also provides a theoretical base for musical feeding to enhance animal welfare through improving the animal growth environment.

## Supplementary Information

Supplementary Information.

## Data Availability

The datasets supporting the original study inferences are included in the study and as additional files. The original sequence data have been uploaded to the Sequence Read Archive (SRA) (NCBI, USA) with the Accession Number: PRJNA901480.
