# Supplementary material for: Music-based interventions in the feeding environment on the gut microbiota of mice
Source: Sci Rep. 2023 Apr 18;13:6313. doi: 10.1038/s41598-023-33522-3 (PMC10111315; doi:10.1038/s41598-023-33522-3)
Supplement: Supplementary file 1 — Supplementary Information. [file 41598_2023_33522_MOESM1_ESM.docx]

Table. S1: The taxonomic analysis of species.

| Sample | Kingdom | Phylum | Class | Order | Family | Genus | Species |
| --- | --- | --- | --- | --- | --- | --- | --- |
| Jcon2 | 40134 | 40134 | 40105 | 39604 | 38286 | 34630 | 14595 |
| Jcon3 | 40134 | 40089 | 40089 | 39315 | 38192 | 33714 | 2434 |
| Jcon4 | 40134 | 40134 | 40039 | 38932 | 37453 | 31368 | 2970 |
| Jcon5 | 40134 | 40134 | 40134 | 39324 | 37997 | 32423 | 10395 |
| Jcon6 | 40134 | 40129 | 40128 | 38822 | 36797 | 30341 | 2761 |
| Jm1 | 40134 | 40129 | 40101 | 39560 | 38122 | 32701 | 5537 |
| Jm2 | 40134 | 40134 | 40134 | 39803 | 38604 | 35343 | 8828 |
| Jm3 | 40134 | 39981 | 39960 | 39261 | 38281 | 31346 | 4220 |
| Jm4 | 40134 | 40134 | 40134 | 39694 | 38880 | 35459 | 532 |
| Jm5 | 40134 | 40134 | 39901 | 38755 | 36336 | 29470 | 2635 |
